# Humanoid Robot Use in Cognitive Rehabilitation of Patients with Severe Brain Injury: A Pilot Study

**DOI:** 10.3390/jcm11102940

**Published:** 2022-05-23

**Authors:** Francesco Corallo, Giuseppa Maresca, Caterina Formica, Lilla Bonanno, Alessia Bramanti, Nicholas Parasporo, Fabio Mauro Giambò, Maria Cristina De Cola, Viviana Lo Buono

**Affiliations:** 1 IRCCS Centro Neurolesi Bonino-Pulejo, 98124 Messina, Italy; francesco.corallo@irccsme.it (F.C.); giuseppa.maresca@irccsme.it (G.M.); katia.formica@irccsme.it (C.F.); lilla.bonanno@irccsme.it (L.B.); nicholas.parasporo@irccsme.it (N.P.); fabio.giambo@irccsme.it (F.M.G.); viviana.lobuono@irccsme.it (V.L.B.); 2 Department of Medicine, Surgery and Dentistry—Medical School of Salerno, University of Salerno, 84084 Fisciano, Italy; alessia.bramanti@gmail.com

**Keywords:** cognitive rehabilitation, humanoid robot, severe acquired brain injury, robotic treatment, cognitive training

## Abstract

Severe acquired brain injury (SABI) is a major global public health problem and a source of disability. A major contributor to disability after SABI is limited access to multidisciplinary rehabilitation, despite evidence of sustained functional gains, improved quality of life, increased return to work, and reduced need for long-term care. Twelve patients with a diagnosis of SABI were enrolled and equally divided into two groups: experimental and control. Patients in both groups underwent intensive neurorehabilitation according to the severity of their disabilities (motor, psycho-cognitive, and sensory deficits). However, in the experimental group, the treatment was performed by using a humanoid robot. At baseline, the two groups differed significantly only in Severe Impairment Battery (SIB) scores. Results showed that the experimental treatment had a higher effect than the traditional one on quality of life and mood. In conclusion, this pilot study provides evidence of the possible effects of relational and cognitive stimulation in more severely brain-injured patients.

## 1. Introduction

Severe acquired brain injury (SABI) is a neurological condition due to severe brain damage followed by a coma of at least 24 h, a Glasgow Coma Scale of eight or less, and/or complex neurological disabilities treatable only in high specialty neurorehabilitation units [1]. SABI includes a variety of traumatic or non-traumatic acute brain lesions, such as stroke and hypoxic-ischemic encephalopathy after cardiac arrest, and cause motor, sensory, cognitive, and/or behavioral impairments [2,3].

The main cognitive consequences of SABI include neuropsychological and psychological symptoms such as spatial–temporal disorientation, a deficit in attention and memory, judgment and abstract thinking, language disorders, personality changes, and alteration in behavioral and emotional functioning (e.g., impulsiveness, anger/irritability, anxiety, fear, and sadness) [4]. SABI impacts the life of an individual and his/her family and represents a significant health and social problem that affects the clinical outcome and quality of life (QoL) of survivors [5]. Although improvements in emergency care have led to increased survival rates, the provision of post-acute neurorehabilitation is inadequate, and the reintegration of survivors into society is limited by the consequences of acquired brain injury [6]. The appropriate management of a patient with SABI requires the intervention of a multidisciplinary and multi-professional team. The primary goal of rehabilitation in hospitalized patients with severe impairment of consciousness is to enable functional recovery that minimizes the impact of residual impairments on the patient’s QoL [7].

Over the last years, the development of new technologies in the field of cognitive rehabilitation has led to the increasing use of computer-based cognitive tools in patients with SABI [8]. Recently, rehabilitation robotics has provided promising training and assistance approaches to mitigate cognitive deficits and reduce anxiety and depressive states in patients with neurological pathologies [9]. The use of robotic devices allows several advantages, including a smaller workforce, longer and more intense exercise compared to traditional treatments, an objective and quantitative assessment of disability, which can be monitored over time, and the possibility of multisensory stimulation of the patient. In particular, patients with traumatic brain injury undergoing robotic treatment plus virtual reality have achieved a greater increase in cognitive flexibility and attention shifting, as well as in executive and visuospatial skills needed to plan and manage daily life [10].

Robots can be used in various daily scenarios or to support motor functions, training, and rehabilitation. There are two main categories of robotic rehabilitation systems, namely therapy robots (divided into physical therapy and emotional therapy robots) and assistive robots (described as physical/social interaction robots) [11]. Recently, human robots have been effectively used in dementia care, and several commercially available robots have been employed with satisfactory results in cognitive stimulation and memory training [12,13].

According to the literature, humanoid robots, known as social robots, have a humanlike appearance in that they have human bodies and behavioral characteristics that mimic human behavior: verbal and non-verbal behavioral cues such as gaze and gestures, body postures, facial emotions, proxemics, etc. [14]. Meanwhile, in rehabilitation, humanoid robots also refer to Socially Assistive Robots (SARs), which assist patients via social interaction and provide innovative tools for rehabilitation [15]. SARs refers to robots intended to assist people primarily in social interactions (e.g., speaking, driving, remembering, observing, and entertaining). Early studies have shown that SARs have the advantage of enhancing mood, social relationships among patients, and emotional expression of individual dementia sufferers [16,17].

In this study, we have described the effects of neurocognitive training performed by using the humanoid robot PEPPER on cognitive and emotive processes, communication, and social skills, in comparison with traditional cognitive treatment, in a cohort of SABI patients.

## 2. Materials and Methods

### 2.1. Study Design and Participants

This pilot study included twelve patients with a diagnosis of SABI who were admitted to the Rehabilitation Unit for SABI of the IRCCS Centro Neurolesi “Bonino-Pulejo” of Messina (Italy) from December 2020 to December 2021. The diagnosis of SABI referred to an episode of traumatic or vascular etiology, with a variety of neurologic symptoms related to alterations in cognition, affectivity, and sensorimotor ability, as well as deficits in the domains of attention and memory.

Subjects were selected according to the following inclusion criteria: (i) SABI diagnosis; (ii) age over 17 years; (iii) level of cognitive functioning (LCF) ≥ 3; Mini-Mental Status Examination (MMSE ≥ 16 ≤24). Exclusion criteria were as follows: (1) vision/hearing loss that limits the participant’s understanding of instructions, or; (2) global aphasia.

All patients were taking drug therapy with antiepileptic, antihemorrhagic, and antihypertensive medications. However, patients were not subjected to further experimental treatments during the study (e.g., brain stimulation, sensory therapy, etc.).

Participants performed a long-term intensive rehabilitative treatment. Before beginning, the sample of enrolled patients was randomly assigned to two groups: experimental group (EG: *n* = 6, submitted to a robotic rehabilitation) and control group (CG: *n* = 6, receiving traditional rehabilitation). Patients did not receive any other cognitive treatment. A more detailed description of the two groups is in Table 1. The patients included in the protocol underwent intensive neurorehabilitation according to the severity of their disabilities (motor, psycho-cognitive, sensory deficit). Thus, patients received conventional physiotherapy, speech therapy, and cognitive rehabilitation. Notably, cognitive rehabilitation was carried out 3 times a week for 8 weeks, and each session lasted about 60 min: the CG group underwent traditional cognitive rehabilitation (sensorial stimulations, pencil-and-paper exercises), while the EG group performed cognitive robot rehabilitation (i.e., PEPPER; Softbank Robotics Aldebaran). The study protocol was approved by the Local Ethics Committee according to the Declaration of Helsinki, register number 23/2020. Caregivers of all patients provided written consent to the study.

### 2.2. Randomization

In order to control potential confounding factors, we randomized the assignment of study subjects between the EG and CG groups. Notably, patients were stratified with respect to gender and age and randomly assigned to a group in a ratio of 1:1.

Given the substantial difference in treatment performance, the randomization was not blinded for participants and care providers. However, study physicians performing the assessment did not know to which treatment the patient has been assigned.

### 2.3. Outcome Measures

The cognitive and behavioral assessment of patients was performed on admission (T0), after 1 month (T1), and after an additional 2 months (T2), i.e., one month after the end of the rehabilitative treatment. The specific psychometric battery was administered by a skilled neuropsychologist, and it included: Level of Cognitive Functioning Scale (LCF); scores ranging from 1 (non-responders) to 8 (purposeful-appropriate person) [18]; MMSE to measure global cognitive status, which maximum score is 30 (a score of 25 or lower is indicative of cognitive impairment [19]); Severe Impairment Battery (SIB), which score ranges from 0 to 100: the severity of impairment is assessed by scores less than 63 [20]; Beck Depression Inventory (BDI-II) [21] and Hamilton Rating Scale for anxiety (HAM-A) [22], used to assess levels of depression and anxiety, respectively, where the highest score corresponds to greater impairment of mood. The neuropsychologist also administered the Functional Independence Measure scale (FIM), which score ranges from 1 (total dependence) to 7 (complete independence) [23], and the EuroQol-5D (EQ-5D) which score ranges from 0 (the worst possible health status) to 100 (the best possible health status) [24].

### 2.4. Robotic Rehabilitation

PEPPER is an industrially produced humanoid robot able to exhibit body language, perceive and interact with its surroundings, and move around. It can also analyze people’s expressions and voice tones, using the latest advances and proprietary algorithms in voice and emotion recognition to trigger interactions. The robot is equipped with features and high-level interfaces for multimodal communication with the humans around it. The touch screen on his chest displays content to highlight messages and support speech. The robot provides feedback by a combination of verbal response and visual feedback, which is displayed on the robot’s tablet screen. The robot’s responses are further accompanied by head and arm gestures (e.g., nodding, clapping, or dancing a victory dance).

Each cognitive session (both traditional and virtual reality) of the experimental treatment included the stimulation of specific cognitive domains: memory, attention, language, spatiotemporal orientation, planning, reasoning, and other executive functions, calculation, and practice. Exercises were parameterized by setting certain robot parameters such as level of difficulty, duration, etc.

The results of each exercise were aggregated according to the objective and stored within a “cloud” service, in a database also containing the patient’s biographical data, the rehabilitation objectives defined by the practitioner, and the results of the related exercises. Patient’s information was only accessed by the operator via tablet.

In most cases, the exercises included an initial phase of exposition of the topic through the robot and a series of successive quizzes, whose possible answers were displayed on the tablet. The robot acquired the answers (right or wrong), the response time, the time of execution of the entire exercise, and the number of attempts (if the exercise provides for it). By starting from this information, an evaluation in percentage was deduced.

### 2.5. Traditional Rehabilitation

The traditional rehabilitative program was planned according to a predefined scheme. Each rehabilitative session was composed as follows: space–time orientation exercises (20 min), attention exercises (30 min), rest (10 min), and memory exercises (30 min). Concerning memory, internal and external aids such as a clock, city maps (for spatial orientation), diaries, notebooks, family photographs for recovering crucial events of the patient’s life (episodic memory), image–word associations to facilitate semantic memory, auditory and visual barrage tasks for visual sustained attention recovery were used. On the whole, exercise difficulty gradually increased during the rehabilitative sessions.

### 2.6. Statistical Analysis

Data were analyzed using the R version 4.0.5 at a 95% confidence level and considered a *p* < 0.05 as statistically significant. Because of the reduced sample dimensionality, a no-parametric approach was performed. Thus, differences between groups at baseline were assessed by the Mann–Whitney U test, whereas proportions by the Chi-squared test.

The Levene test was used to assess homoscedasticity before using the lme4 package of R to perform a linear mixed effects analysis of the relationship between clinical outcome and treatment. We included in the model the two levels variable ‘group’ (EG = experimental group; CG = control group) and the three-level variable ‘evaluation time’ (T0, T1, T2) as fixed effects. Subject’s variability was considered as a random effect by including correlated intercepts and slopes for the fixed factors. The interaction between the fixed effects was also considered. *p*-values were obtained by likelihood ratio tests of the full model (full model) compared to the model without ‘group’ as fixed effect (WG model). A random effects analysis of variance model was used to estimate the Intraclass correlation (ICC).

## 3. Results

At baseline, the two groups significantly differed only in SIB scores (*p* = 0.04), as visible in Table 1.

Significant differences between the WG models and the full models were found for the following outcomes: HAM-A (X2(3) = 53.62; *p* < 0.001), EQ-5D (X2(3) = 101.27 *p* < 0.001), and BDI-II (X2(3) = 57.99; *p* < 0.001). In addition, for these measures, the full models had lower AIC and BIC values and considerably reduced deviations than the WG models, as shown in Table 2.

The ICCs of any model showed a high correlation between two evaluations on the same patient and at the same time: 0.76 for HAM-A, 0.87 for EQ-5D, and 0.84 for BDI-II (Table 3). Indeed, the interaction between the fixed effects was significant in such models. In particular, the interaction group: time significantly affected the patients’ scores from baseline to T1 for EQ-5D (t = 10.65, *p* < 0.001) and BDI-II (t = 6.50, *p* < 0.001); whereas from baseline to T2, the interaction significantly affected the patients’ scores of all these three outcomes, indicating that changes between the two groups increased over time in such measures. Results of the mixed effects model reported in Table 3 also showed that the experimental treatment had a higher effect than the traditional one on QoL. Indeed, for EQ-5D, we observed a mean score in EG lower by 1.50 ± 0.73 than in CG (t = −2.04, *p* = 0.04).

**Figure 1:**
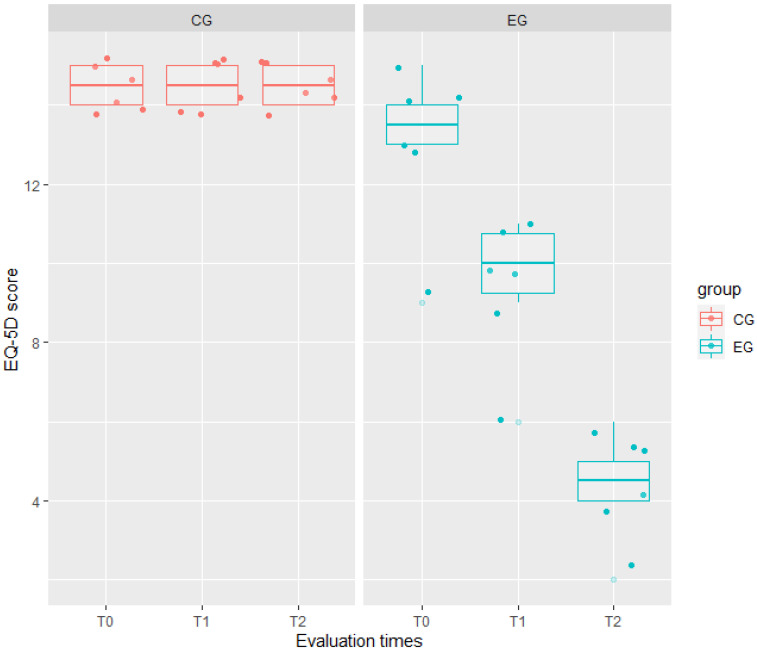
Comparison of quality of life scores (measured by EQ-5D = EuroQol-5D scale) between the experimental group (EG) in green, and the control group (CG) in red, at any evaluation time (T0, T1, and T2). Any box represents data distribution: the bounds at the top and bottom are the first and third quartiles, the center line is the median value, the whiskers from the box indicate the minimum and maximum values, dark (jittered) points represent patient EQ-5D scores, while clear points represent outliers. CG boxplots are similar among evaluation times, while EG boxplots show a significant decrease from T0 to T2.

**Figure 2:**
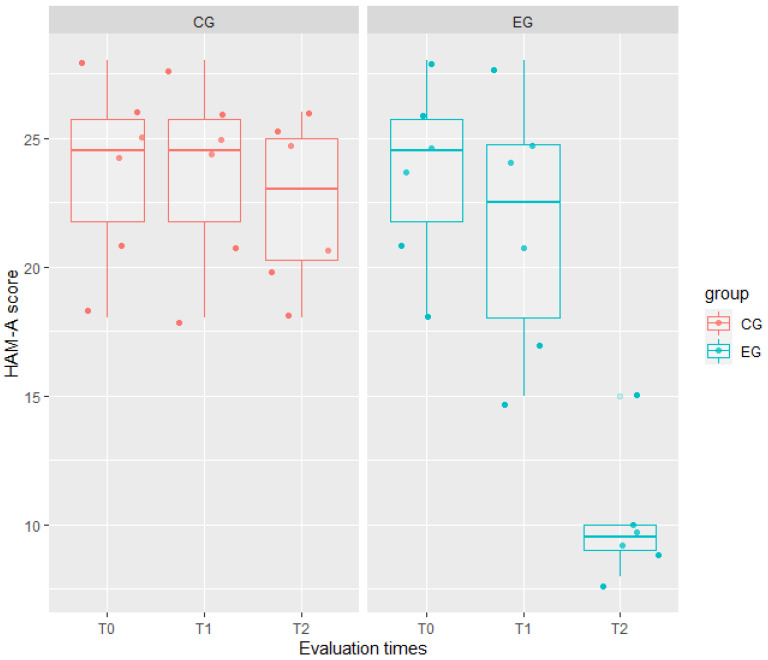
Comparison of anxiety scores (measured by HAM-A = Hamilton Rating Scale) between the experimental group (EG) in green, and the control group (CG) in red, at any evaluation time (T0, T1, and T2). Any box represents data distribution: the bounds at the top and bottom are the first and third quartiles, the center line is the median value, the whiskers from the box indicate the minimum and maximum values, dark (jittered) points represent patient HAM-A scores, while clear points represent outliers. From T0 to T1, CG boxplots are similar while EG boxplots show a decrease in median score, more apparent from T1 to T2.

**Figure 3:**
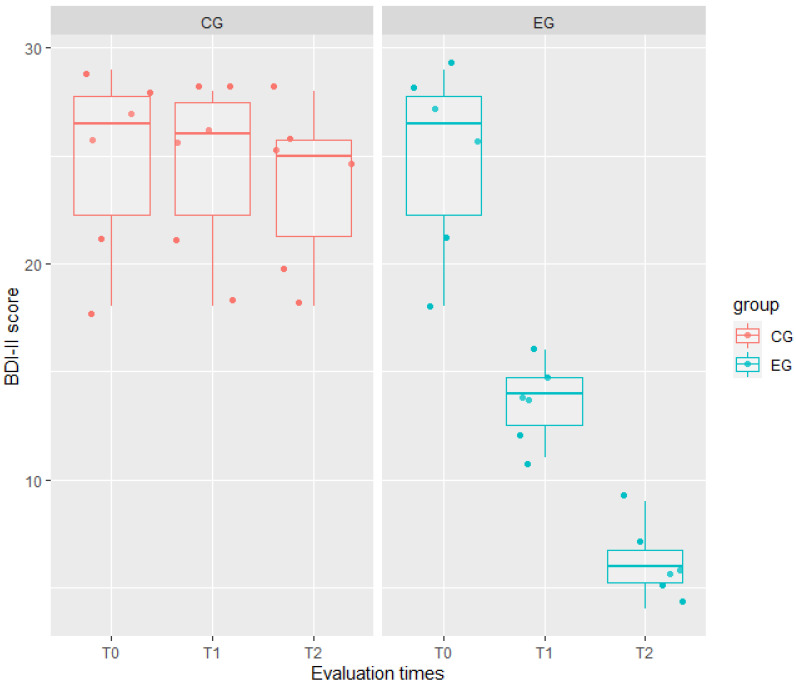
Comparison of depression scores (measured by BDI-II = Beck Depression Inventory) between the experimental group (EG) in green, and the control group (CG) in red, at any evaluation time (T0, T1, and T2). Any box represents data distribution: the bounds at the top and bottom are the first and third quartiles, the center line is the median value, the whiskers from the box indicate the minimum and maximum values, dark (jittered) points represent patient BDI-II scores, while clear points represent outliers. CG boxplots show a not significant decrease among evaluation times, while EG boxplots show a significant decrease from T0 to T2.

Moreover, the experimental treatment significantly affected the mood of EG patients, by decreasing from baseline to T2 both the HAM-A (−12.33 ± 1.37, t = 8.98, *p* < 0.001) and BDI-II (−17.50 ± 1.67, t = −10.50, *p* < 0.001) scores, as visible in Figure 1, Figure 2 and Figure 3.

## 4. Discussion

The results of this preliminary study were very surprising, as unlike what might have been expected, patients in the experimental group did not improve their cognitive performance after the rehabilitation program with the humanoid robot, but they perceived better quality of life and better mood than the control group.

We can hypothesize that the use of the robot has contributed substantially for several reasons. First, the robot lightens the burden of cognitive rehabilitation and decreases frustration levels. Second, the reactions of the robot are directly related to the input provided by the patient. Thus, the relational aspect requires a greater effort. Finally, the play-therapeutic aspect plays an important role in mood. Unfortunately, to date, there is not enough literature to support our hypothesis because studies have focused on patients with different neurological disorders. Indeed, in [25], the authors used the humanoid robot Pepper within a training program aimed to improve the cognitive status of people with dementia, investigating how patients relate to the robot and perceive the serious games it is equipped with [26]. In this study, it was observed that the elderly engaged more with the robot from one session to the next, showing a positive perception of interaction with it. In several studies, it has been argued that humanoid robots are a suitable tool for use with dementia patients, as well as with relatives and caregivers and that their presence brings patients with dementia in a more positive emotional state [27,28]. In particular, music sessions stimulate patients to recall memories and talk about their past [29].

Cifuentes et al. [30] explored the implications of social robots in healthcare scenarios, and they conducted a review study on the applications of social robots, including addressing their perception and acceptance by children and adults. This review revealed that adults and children who were exposed to an intervention with social robots improved in social connection and communication, as well as their mood, and showed a decrease in depression, anxiety, and fear. Similarly, Kabacinska et al. [31] conducted a literature survey on how social robots have been used as means to support mental health in children. The study findings suggested that interventions with social robots have a positive impact on reducing stress and improving levels of positive affect.

This study has some limitations, including the small sample size, lack of design control for the presence of pharmacological treatments, and lack of unambiguous training assessments and uncontrollable factors. On the other hand, the small number of participants is the most common challenge facing cognitive training researchers in this field. This challenges the generalization and reliability of the experimental results reported in this study and may account for the significant difference in SIB scores at baseline between the two groups.

## 5. Conclusions

Robotic rehabilitation has provided promising assistive approaches to mitigate cognitive deficits. The field of SABI is still being studied because there are no standardized protocols [32]. In this paper, we presented the results of an experimental study carried out in the context of rehabilitation interventions aimed at improving cognitive performance in SABI patients. Currently, there are several challenges in using humanoid robots for cognitive rehabilitation. Chief among them are ethical issues, robot reliability, appropriated user-centered (or stakeholder-centered) treatment design, customization of the robot-assisted cognitive training system, and cost-effectiveness. Future research must also consider human-robot collaboration and social cognition to facilitate a natural human-robot interaction. Probably, the most common challenge faced by researchers of cognitive training is the small size of participants. This challenges the generalization and reliability of experimental results.

## Figures and Tables

**Table 1:** Descriptive analysis of the sample’s characteristics at baseline.

	All	EG	CG	*p*-value
**Participants**	12	6	6	-
**Age** (*years*)	46.9 ± 10.7	46.7 ± 10.9	47.2 ± 11.4	0.99
**Education** (*years*)	11.3 ± 2.5	11.3 ± 2.6	11.3 ± 2.6	0.99
**Males**	7 (58.3)	3 (50.0)	4 (66.7)	0.99
**Side of the lesion—Bilateral**	8 (66.7)	3 (50.0)	5 (83.3)	0.54
**Etiology—Traumatic**	7 (58.3)	3 (50.0)	4 (66.7)	0.99
**SIB**	59.2 ± 6.4	59.8 ± 7.5	58.7± 5.9	0.04
**LCF**	4.1 ± 0.3	4.0 ± 0.0	4.2 ± 0.4	0.40
**MMSE**	17.8 ± 3.9	17.8 ± 4.1	17.8 ± 4.1	0.99
**HAM-A**	23.7 ± 3.4	23.7 ± 3.6	23.7 ± 3.6	0.99
**FIM**	81.7 ± 5.4	81.7 ± 5.7	81.7 ± 5.7	0.99
**EQ-5D**	13.7 ± 1.7	13.0 ± 2.1	14.5 ± 0.6	0.09
**BDI-II**	24.8 ± 4.1	24.8 ± 1.7	24.8 ± 4.4	0.99

Legend: SIB: Severe Impairment Battery; LCF = Level of Cognitive Functioning Scale; MMSE = Mini Mental State Examination; HAM-A = Hamilton Rating Scale for anxiety; BDI-II = Beck Depression Inventory; FIM = Functional Independence Measure scale; EQ-5D = EuroQol-5D; EG = Experimental group; CG = Control group. Mean ± standard deviation was used to describe continuous variables; proportions (numbers and percentages) were used to describe categorical variables.

**Table 2:** Comparison between the full model (full model) and model without ‘group’ as fixed effect (WG model), for each outcome measure.

Outcome Measure		AIC	BIC	Deviance	Chi-Square	df	*p*-Value
**SIB**	WG model Full model	176.30 176.45	187.39 192.29	162.30 156.45	5.85	3	0.119
**LCF**	WG model Full model	59.36 64.46	70.45 80.29	45.36 44.46	0.90	3	0.825
**MMSE**	WG model Full model	112.96 115.36	124.04 131.20	98.96 95.36	3.59	3	0.309
**HAM-A**	WG model Full model	224.81 177.20	235.90 193.03	210.81 157.20	53.62	3	<0.001
**FIM**	WG model Full model	216.00 221.05	227.09 236.88	202.00 201.05	0.95	3	0.813
**EQ-5D**	WG model Full model	183.92 88.65	195.01 104.49	169.92 68.65	101.27	3	<0.001
**BDI-II**	WG model Full model	239.42 187.42	250.51 203.26	225.42 167.42	57.99	3	<0.001

Legend: SIB: Severe Impairment Battery; LCF = Level of Cognitive Functioning Scale; MMSE = Mini Mental State Examination; HAM-A = Hamilton Rating Scale for anxiety; BDI-II = Beck Depression Inventory; FIM = Functional Independence Measure scale; EQ-5D = EuroQol-5D; AIC = Akaike’s Information criteria; BIC = Bayesian information criteria; df = degrees of freedom.

**Table 3:** Results of the mixed effects analysis performed on the outcome measures HAM-A, BDI-II, and EQ-5D: (a) Fixed effects; (b) Random effects.

Outcome Measure		Coeff. Estimate	Std. Err.	*t*-Value	*p*-Value	ICC
**HAM-A**	EG	<0.001	0.97	0.00	1.000	0.76
	T1	<0.001	0.97	0.00	1.000	
	T2	−1.17	0.97	1.20	0.239	
	EG × T1	−2.00	1.37	1.45	0.156	
	EG × T2	−12.33	1.37	8.98	<0.001	
**EQ-5D**	EG	−1.50	0.73	2.04	0.041	0.87
	T1	<0.001	0.23	0.00	1.000	
	T2	<0.001	0.23	0.00	1.000	
	EG × T1	−3.50	0.33	10.65	<0.001	
	EG × T2	−8.67	0.33	26.37	<0.001	
**BDI-II**	EG	<−0.001	1.72	0.00	1.000	0.84
	T1	−0.33	1.18	0.28	0.779	
	T2	−1.17	1.18	0.99	0.330	
	EG × T1	−10.83	1.67	6.50	<0.001	
	EG × T2	−17.50	1.67	10.50	<0.001	
(a)						
**Outcome measure**			**Variance**	**Std. Dev.**	**Correlation**	
**HAM-A**	Subj (Intercept)		8.85	2.97	−1.00	
	Subj (EG)		0.03	0.16		
	Residual		2.83	1.68		
**EQ-5D**	Subj (Intercept)		0.19	0.44	−0.44	
	Subj (EG)		2.92	1.71		
	Residual		0.16	0.40		
**BDI-II**	Subj (Intercept)		12.48	3.53	−1.00	
	Subj (EG)		9.33	3.05		
	Residual		4.16	2.04		
(b)						

Legend: HAM-A = Hamilton Rating Scale for anxiety; BDI-II = Beck Depression Inventory; EQ-5D = EuroQol-5D; EG = Experimental Group.

## Data Availability

Not applicable.

## References

[1] (1998). Lineeguida del Ministro della Sanità per le Attività di Riabilitazione. https://www.gazzettaufficiale.it/eli/id/1998/05/30/098A4518/sg.

[2] Lorenz L.S., Doonan M. (2021). Value and Cost Savings from Access to Multi-disciplinary Rehabilitation Services After Severe Acquired Brain Injury. Front. Public Health.

[3] Calabrò R.S., Bramanti A., Garzon M., Celesti A., Russo M., Portaro S., Naro A., Manuli A., Tonin P., Bramanti P. (2018). Telerehabilitation in individuals with severe acquired brain injury: Rationale, study design, and methodology. Medicine.

[4] Castellani G.B., Miccoli G., Cava F.C., Salucci P., Colombo V., Maietti E., Palandri G. (2022). From Shunt to Recovery: A Multidisciplinary Approach to Hydrocephalus Treatment in Severe Acquired Brain Injury Rehabilitation. Brain Sci..

[5] Formisano R., Carlesimo G.A., Sabbadini M., Loasses A., Penta F., Vinicola V., Caltagirone C. (2004). Clinical predictors and neuropsychological outcome in severe traumatic brain injury patients. Acta Neurochir..

[6] D’Ippolito M., Aloisi M., Azicnuda E., Silvestro D., Giustini M., Verni F., Formisano R., Bivona U. (2018). Changes in Caregivers Lifestyle after Severe Acquired Brain Injury: A Preliminary Investigation. Biomed. Res. Int..

[7] Menon D.K., Bryant C. (2019). Time for change in acquired brain injury. Lancet Neurol..

[8] De Luca R., Calabrò R.S., Bramanti P. (2018). Cognitive rehabilitation after severe acquired brain injury: Current evidence and future directions. Neuropsychol. Rehabil..

[9] Manuli A., Maggio M.G., Latella D., Cannavò A., Balletta T., De Luca R., Naro A., Calabrò R.S. (2020). Can robotic gait rehabilitation plus Virtual Reality affect cognitive and behavioural outcomes in patients with chronic stroke? A randomized controlled trial involving three different protocols. J. Stroke Cerebrovasc. Dis..

[10] Yuan F., Klavon E., Liu Z., Lopez R.P., Zhao X. (2021). A Systematic Review of Robotic Rehabilitation for Cognitive Training. Front. Robot. AI.

[11] Yakub F., Khudzari A.Z.M., Mori Y. (2014). Recent trends for practical rehabilitation robotics, current challenges and the future. Int. J. Rehabil. Res..

[12] De Carolis B., Carofiglio V., Grimandli I., Macchiarulo N., Palestra G., Pino O. Using the pepper robot in cognitive stimulation therapy for people with mild cognitive impairment and mild dementia. Proceedings of the ACHI-The Thirteenth International Conference on Advances in Computer-Human Interactions.

[13] Nocentini O., Fiorini L., Acerbi G., Sorrentino A., Mancioppi G., Cavallo F. (2019). A Survey of Behavioral Models for Social Robots. Robotics.

[14] Chuah S.H.W., Yu J. (2021). The future of service: The power of emotion in human-robot interaction. J. Retail. Consum. Serv..

[15] Biffi E., Beretta E., Storm F.A., Corbetta C., Strazzer S., Pedrocchi A., Ambrosini E. (2021). The Effectiveness of Robot- vs. Virtual Reality-Based Gait Rehabilitation: A Propensity Score Matched Cohort. Life.

[16] Koutentakis D., Pilozzi A., Huang X. (2020). Designing Socially Assistive Robots for Alzheimer’s Disease and Related Dementia Patients and Their Caregivers: Where We are and Where We are Headed. Healthcare.

[17] Wang W.S., Mendonca R., Kording K., Avery M., Johnson M.J. (2019). Towards Data-Driven Autonomous Robot-Assisted Physical Rehabilitation Therapy. IEEE Int. Conf. Rehabil. Robot.

[18] Gouvier W.D., Blanton P.D., LaPorte K.K., Nepomuceno C. (1987). Reliability and validity of the Disability Rating Scale and the Levels of Cognitive Functioning Scale in monitoring recovery from severe head injury. Arch. Phys. Med. Rehabil..

[19] Kang Y., NA D.L., Hahn S. (1997). A validity study on the Korean Mini-Mental State Examination (K-MMSE) in dementia patients. J. Korean Neurol. Assoc..

[20] Panisset M., Roudier M., Saxton J., Boiler F. (1994). Severe impairment battery: A neuropsychological test for severely demented patients. Arch. Neurol..

[21] Whisman M.A., Perez J.E., Ramel W. (2000). Factor structure of the Beck Depression Inventory—Second Edition (BDI-II) in a student sample. J. Clin. Psychol..

[22] Maier W., Buller R., Philipp M., Heuser I. (1998). The Hamilton Anxiety Scale: Reliability, validity and sensitivity to change in anxiety and depressive disorders. J. Affect. Disord..

[23] Hamilton B.B., Laughlin J.A., Fiedler R.C. (1994). Interrater reliability of the 7-level Functional Independence Measure (FIM). Scand. J. Rehabil. Med..

[24] Balestroni G., Bertolotti G. (2012). L’EuroQol-5D (EQ-5D): Uno strumento per la misura della qualità della vita [EuroQol-5D (EQ-5D): An instrument for measuring quality of life. Monaldi. Arch. Chest Dis..

[25] Manca M., Paternò F., Santoro C., Zedda E., Braschi C., Franco R., Sale A. (2021). The impact of serious games with humanoid robots on mild cognitive impairment older adults. Int. J. Hum. Comput. Stud..

[26] Osaka K., Tanioka R., Betriana F., Tanioka T., Kai Y., Locsin R.C. (2021). Robot Therapy Program for Patients with Dementia: Its Framework and Effectiveness. Information Systems: Intelligent Information Processing Systems, Natural Language Processing, Affective Computing and Artificial Intelligence, and an Attempt to Build a Conversational Nursing Robot.

[27] Wu Y.H., Fassert C., Rigaud A.S. (2012). Designing robots for the elderly: Appearance issue and beyond. Arch. Gerontol. Geriatr..

[28] Sumioka H., Shiomi M., Honda M., Nakazawa A. (2021). Technical Challenges for Smooth Interaction with Seniors with Dementia: Lessons from Humanitude™. Front. Robot. AI..

[29] Zuschnegg J., Paletta L., Fellner M., Steiner J., Pansy-Resch S., Jos A., Koini M., Prodromou D., Halfens R.J.G., Lohrmann C. (2021). Humanoid socially assistive robots in dementia care: A qualitative study about expectations of caregivers and dementia trainers. Aging Ment. Health.

[30] Cifuentes C.A., Veneman J.F., Rocon E., Rodriguez-Guerrero C. (2022). Editorial: Interfacing Humans and Machines for Rehabilitation and Assistive Devices. Front. Robot. AI.

[31] Kabacińska K., Prescott T.J., Robillard J.M. (2021). Socially assistive robots as mental health interventions for children: A scoping review. Int. J. Soc. Robot..

[32] La Gattuta E., Corallo F., Lo Buono V., De Salvo S., Caminiti F., Rifici C., Alagna A., Arcadi F., Bramanti A., Marino S. (2018). Techniques of cognitive rehabilitation in patients with disorders of consciousness: A systematic review. Neurol. Sci..

